# A novel portable flip-phone based visual behaviour assay for zebrafish

**DOI:** 10.1038/s41598-023-51001-7

**Published:** 2024-01-02

**Authors:** Vanessa Rodwell, Annabel Birchall, Ha-Jun Yoon, Helen J. Kuht, William H. J. Norton, Mervyn G. Thomas

**Affiliations:** 1 The University of Leicester Ulverscroft Eye Unit, School of Psychology and Vision Sciences, University of Leicester, RKCSB, PO Box 65, Leicester, LE2 7LX UK; 2 Department of Genetics and Genome Biology, University of Leicester, Leicester, LE1 7RH UK

**Keywords:** Animal behaviour, Developmental biology, Neuroscience, Zoology, Behavioural methods, Biological models, Experimental organisms, High-throughput screening

## Abstract

The optokinetic reflex (OKR) serves as a vital index for visual system development in early life, commonly observed within the first six months post-birth in humans. Zebrafish larvae offer a robust and convenient model for OKR studies due to their rapid development and manageable size. Existing OKR assays often involve cumbersome setups and offer limited portability. In this study, we present an innovative OKR assay that leverages the flexible screen of the Samsung Galaxy Z Flip to optimize setup and portability. We conducted paired slow-phase velocity measurements in 5-day post-fertilization (dpf) zebrafish larvae (n = 15), using both the novel flip-phone-based assay and a traditional liquid–crystal display (LCD) arena. Utilizing Bland–Altman plots, we assessed the agreement between the two methods. Both assays were efficacious in eliciting OKR, with eye movement analysis indicating high tracking precision in the flip-phone-based assay. No statistically significant difference was observed in slow-phase velocities between the two assays (p = 0.40). Our findings underscore the feasibility and non-inferiority of the flip-phone-based approach, offering streamlined assembly, enhanced portability, and the potential for cost-effective alternatives. This study contributes to the evolution of OKR assay methodologies, aligning them with emerging research paradigms.

## Introduction

The optokinetic reflex (OKR) is an essential mechanism for stabilizing gaze and optimizing visual input, emerging within the first six months of a child's development^1^. The reflex is induced by dynamic visual stimuli that occupy a considerable portion of the visual field. This behaviour consists of two distinct eye movement phases: the 'slow phase,' which follows the stimulus to minimize retinal slip, and the 'fast phase,' which repositions the eyes in the opposite direction^1^.

Zebrafish (*Danio* *rerio*) offer a powerful model for investigating sensory processing, given their rapid maturation, cost-effective maintenance, and high-throughput assay compatibility^2–5^. Zebrafish develop a fully functional OKR as early as 3–5 days post-fertilization (dpf). These attributes, alongside a regulatory environment that often classifies zebrafish larvae up to 5 dpf as 'unprotected organisms' in certain jurisdictions like the UK, facilitate streamlined research. Notably, zebrafish share substantial gene homology with humans, particularly in genes related to disease, thus serving as effective models for translational research^6^.

Various visual behavioural assays have been developed for zebrafish, including the light–dark preference test for stress response^7^, social interaction tests for autism research^8^, and the startle response for sensorimotor gating studies^9^. Among these, both the optomotor response (OMR) and OKR assays have gained prominence for studying vision-related behaviours^4,10^. However, unlike OMR, which examines voluntary swim behaviour, OKR allows for focused study of eye movements, offering a more in-depth examination of retinal function and neural circuitry^11^.

Despite its utility, the OKR assay setup poses logistical challenges. Traditional methods involve complex machinery, such as rotating striped drums, which consume substantial time and laboratory space^12^. While technological advancements have led to more robust setups using mirrors/projectors^13,14^ or compact LCD/LED-based setups^15,16^, these too have limitations. They require significant workspace and present hurdles for smaller research teams, particularly in settings where laboratory space is at a premium.

**Figure 1:**
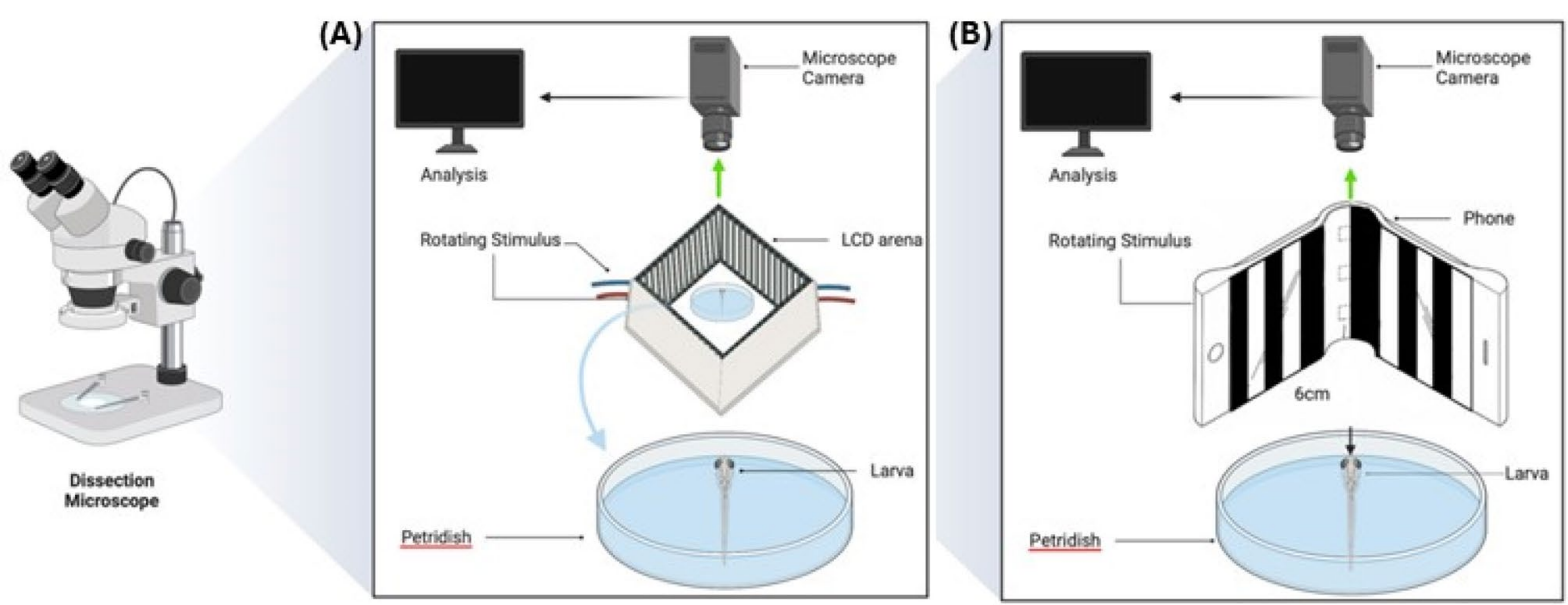
(**A**) LCD based assay setup; (**B**) Flip-phone assay setup.

In light of these considerations, the present study aims to address these logistical constraints by introducing an optimized OKR assay leveraging the flexible screen technology of flip-phones (Fig. 1). We validate the efficacy of this novel setup through a comparative analysis with standard LCD based assays, focusing on the precision of eye-tracking measurements. In this paper we highlight the non-inferiority of the flip-phone-based approach and its implications for streamlined assembly, enhanced portability, and the potential for more accessible and cost-effective visual behaviour studies.

## Results

### Characterization of eye movements

**Figure 2:**
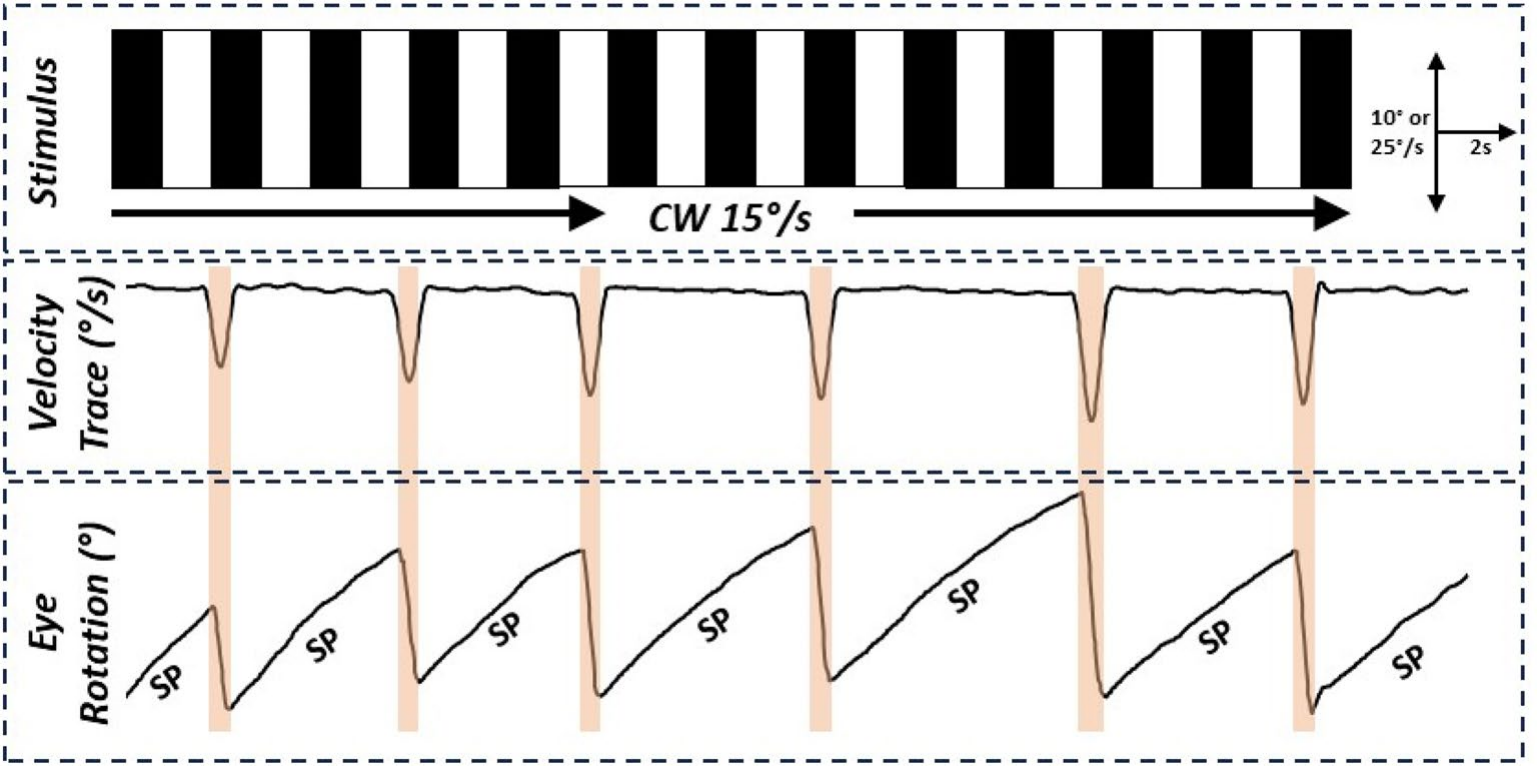
Example eye movement trace shown alongside eye velocity trace. The orange shaded region represents the quick phase. Unshaded regions of the trace represent the slow phases (SP). The SP is characterised by a gradual, steady movement of the eyes as they track a moving visual stimulus. The quick phase, is the rapid, compensatory movement that repositions the eyes in the opposite direction after they reach a certain limit in the SP. This action resets the eye position, allowing the continuation of tracking movement. In our velocity trace, this phase is indicated by sharp spikes (orange shaded region), representing rapid changes in eye position. Upward deflections indicate clockwise (CW) eye rotations, while downward deflections represent counter-clockwise (CCW) rotations. The spatial frequency and speed of the optokinetic stimuli were 0.04 cycles per degree and 15°/s, respectively. Peaks and troughs were detected using custom Python scripts to delineate the quick phases and slow phases (SP) of the zebrafish optokinetic responses.

**Figure 3:**
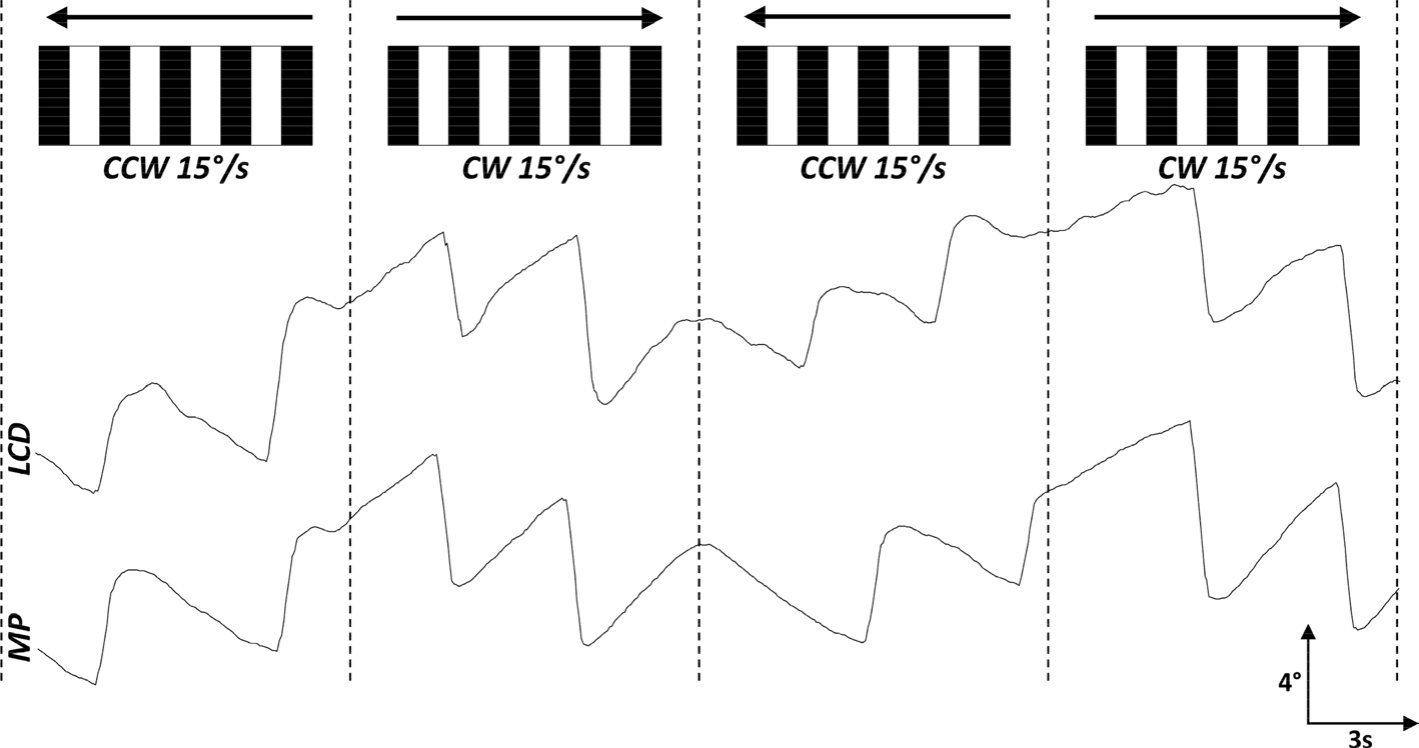
Representative eye movement traces from a wild-type zebrafish when exposed to optokinetic stimuli via LCD and mobile phone (MP) screens. Upward deflections indicate clockwise (CW) eye rotations, while downward deflections represent counter-clockwise (CCW) rotations. The spatial frequency and speed of the optokinetic stimuli were 0.04 cycles per degree and 15°/s, respectively.

Both the LCD-based and the mobile phone-based assays successfully captured the stereotypic eye movement traces associated with OKR, featuring the slow and fast phases. Example of a velocity trace alongside the eye rotation trace with demarcated quick and slow phases are shown in Fig. 2. A visual comparison demonstrated congruency in the eye movement morphology between the two assays (Fig. 3).

### Quantitative assessment of slow-phase velocity

**Figure 4:**
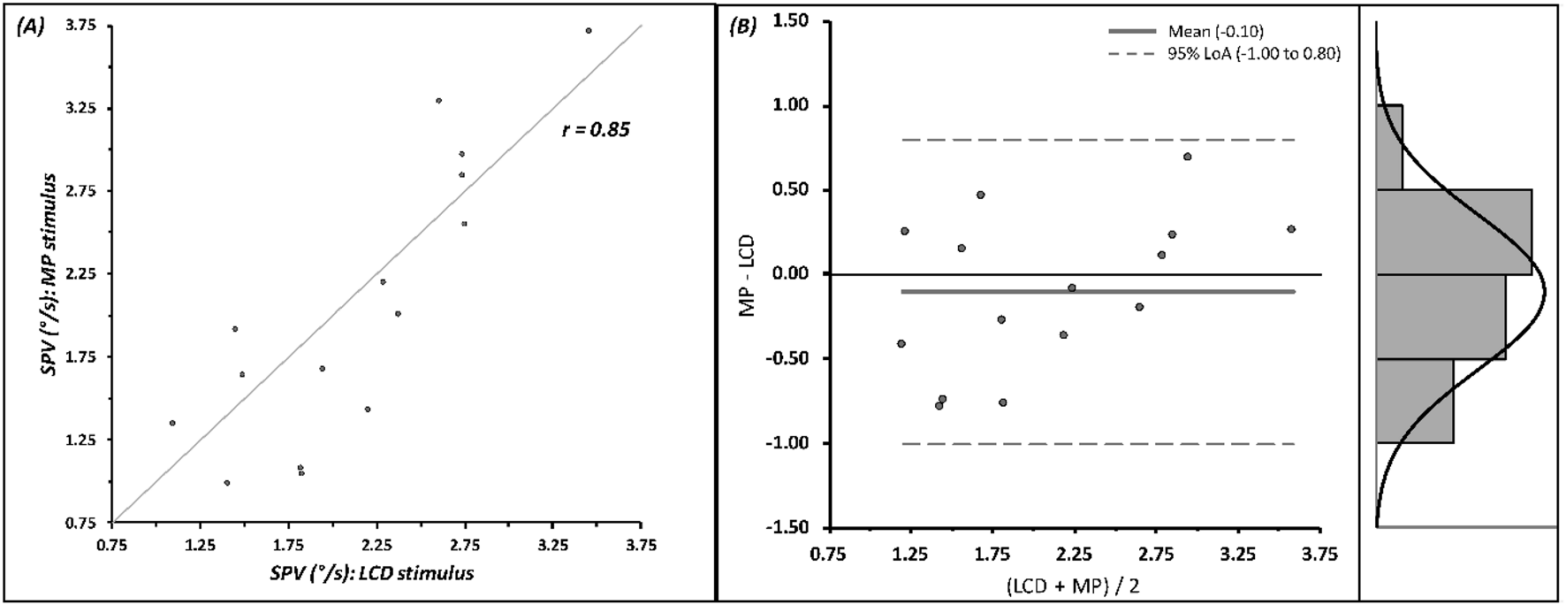
(**A**) Scatter plot representing slow-phase eye velocity (SPV) measurements obtained from zebrafish subjects stimulated via the LCD and mobile phone (MP) assays. (**B**) Bland–Altman plot comparing SPV measurements between the two assays. The solid grey line represents the mean difference, while the dashed grey lines demarcate the 95% limits of agreement (LoA). An accompanying histogram illustrates the distribution of measurements within these limits.

For the LCD-based assay, slow-phase eye velocity ranged from 1.10 to 3.46 deg/s. In contrast, the mobile phone-based assay exhibited a velocity range of 0.99 to 3.59 deg/s. A scatter plot depicting the relationship between the two assays revealed a strong correlation, with a correlation coefficient (r) of 0.85 (Fig. 4A).

### Methodological agreement and statistical comparison

The Bland–Altman plot indicated a mean difference of − 0.10 between the slow-phase eye velocity measurements from both assays. The 95% limits of agreement were − 1.00 to 0.80, highlighting the absence of systematic bias between the methods (Fig. 4B). Statistical comparison revealed no significant difference in slow-phase eye velocities between the assays (p = 0.40).

### Summary of findings

In summary, our data provide robust evidence for the comparability of the flexible mobile phone-based assay with the traditional LCD screen assay in measuring slow-phase eye velocity. The substantial correlation coefficient of 0.85, along with the absence of significant differences (p = 0.40) and lack of systematic bias, as revealed by the Bland–Altman plot, collectively underscore the validity and reliability of the mobile phone-based assay. These findings support the utility of the mobile phone assay as an efficacious alternative for eliciting optokinetic reflex in zebrafish studies.

## Discussion

In this study, we introduced and optimized a novel flip-phone-based assay, utilizing the Samsung Galaxy Z Flip's bendable screen to elicit OKR in larval zebrafish. Our findings show that it not only successfully elicits OKR but also offers high tracking precision. Importantly, when measured against the traditional LCD arena assay, the flip-phone approach yielded comparable results in terms of slow-phase velocities.

Historically, OKR studies have been hindered by the challenges of setting up traditional assays, especially when considering portability. The LCD-based or projector-based assay, the currently preferred technique, has notable limitations in these areas. Our work adds to the literature by demonstrating the feasibility and effectiveness of a more portable and user-friendly approach. This aligns with the evolving research landscape that prioritizes flexibility and adaptability. This trend has been observed in clinical practice in ophthalmology where portable retinal imaging devices are increasingly being introduced allowing more remote examinations and studies to be performed^17^.

The Samsung Galaxy Z Flip phone, used in our novel OKR assay method, was initially the pricier option compared to the LCD assay, which costs around £300 excluding computer/software. However, with other manufacturers producing more affordable flexible smartphones and the rise of refurbished units available for £250–350, the cost difference is considerably narrower now. Additionally, while the LCD assay is solely for research, the flip-phone maintains its primary function as a mobile device. Considering its popularity, researchers might already possess one, further reducing the effective cost with the potential to also use it for quick demonstrations in practical classes. Similarly, it can be used in shared workspaces allowing for flexible working where microscopes and computers may be required for multiple different experiments.

The results have several practical implications. First, the streamlined assembly of the flip-phone assay can reduce setup times, enhancing research efficiency. Although setup time and ease of use were not formal outcome measures, we noted a marked difference in setup duration between the two methods. The traditional LCD screen-based assay required more than 30 minutes to set up, in contrast to the flip-phone assay, which was ready in less than 5 minutes. Additionally, all investigators reported ease in setting up the mobile phone assay, and it proved simple enough for administration by undergraduate medical students (VR and AB). Secondly, the portability inherent to this method broadens the range of potential research settings, making it suitable for field studies or environments with limited resources. In our study we had used fixed viewing angles with the flip-phone, however the flexible screen of the flip-phone allows for adjustments in viewing angles, which can be useful in studies examining the impact of stimulus orientation on visual tracking. This feature is particularly beneficial for experiments that require a range of visual stimuli presentations, which is not as easily achievable with fixed LCD screens. The high-resolution screens with flip-phones has the potential to provide finer control over the stimulus presentation, which maybe important for detailed visual behaviour studies. Moreover, the potential cost-effectiveness of the flip-phone approach could democratize access to OKR studies, allowing for a wider range of institutions to partake in such research.

In our assay we used a Samsung Galaxy Z flip due to its widespread availability and popularity, thus making it a practical choice for broad application in research settings. While our study utilised this specific model, we anticipate that other flip-phones with similar screen technology could be equally suitable for OKR testing. The choice of a 30-degree opening angle was made to stimulate approximately 120 degrees of the larval visual field. This angle represents a balance: a smaller opening would increase the screen's lateral proximity to the larvae, altering the visual angle of the gratings on the retina, potentially impacting the stimulus perception. Conversely, a larger angle would reduce the stimulated visual field, possibly diminishing the efficacy of the OKR response.

The flip-phone assay may have constraints in administering full field OKR stimulation. Notably, distinctions between full field and anterior field visual stimulation have been documented in certain mouse models^18^. In such scenarios, the LCD arena or previously described spherical arena methodologies would be more suitable^15^. Our study was limited to the use of the flip-phone with specific zebrafish larval stage and stimulus patterns. Whilst in principle we anticipate this could be extended to additional developmental stages and other species further optimisation and validation maybe required. Beyond the method itself, investigating its potential for high-throughput screening or integration into larger behavioural study frameworks could be valuable.

In conclusion, our study introduces a novel flip-phone based assay for eliciting OKR in larval zebrafish, showcasing its potential as a viable, efficient, and potentially cost-effective alternative to traditional methods. As the research landscape evolves, tools that offer flexibility, portability, and efficiency will be important, and our findings suggest that the flip-phone approach aligns well with these demands.

## Materials and methods

### Ethical compliance and animal housing

Ethical approval for this study was obtained from the Animal Welfare Ethical Review Body (AWERB) at the University of Leicester. All procedures were conducted in compliance with UK animal welfare regulations, as stipulated by the Animals Scientific Procedures Act (ASPA 2012). Project license was obtained from the UK Home Office (PP1567795). ARRIVE guidelines and zebrafish OKR (ZOK) reporting guidelines^10^ were followed and minimum reporting checklist have been included (supplementary material). Zebrafish were housed at the Pre-Clinical Research Facility (PRF) on the University of Leicester campus, maintained under a 14:10 light–dark cycle. Specific water parameters were controlled, including temperature (28 °C), pH (6.8–8.2), and other water quality metrics (0 ppm ammonia, 0 ppm nitrate, and < 20 ppm nitrite). Standardised stocking density and feeding protocols were maintained at the PRF.

### Larval preparation

Larvae at 5 days post-fertilization (dpf) were embedded in 4% pre-warmed (28 °C) methylcellulose. Larvae were oriented dorsally upward in a 35 mm Petri dish for imaging and stimulus presentation.

### Traditional LCD screen assay

The traditional OKR assay utilized four LCD panels, each measuring 80 mm × 50 mm, arranged at orthogonal angles to form a square-shaped arena. The Petri dish housing the larval zebrafish was positioned 6 cm away from the stimulus (Fig. 1a).

### Novel flip-phone assay

The novel flip-phone OKR assay was conducted under a Leica S9i dissection microscope. The Samsung Galaxy Z Flip was positioned 6 cm away from the larval eyes and opened at a 30-degree angle, covering approximately 120 degrees of the larval visual field (Fig. 1b).

### Stimulus generation

Visual stimuli, consisting of vertical black-and-white sinusoidal gratings, were generated using PsychoPy version 2023.1.0^19^, an open-source Python software. The gratings were presented at 100% contrast and a luminance of 1200 cd/m^2^. The stimulus parameters are detailed in Table 1.

**Table 1:** Stimulus parameters.

Duration (s)	Aspect ratio	Grating orientation	Grating spatial frequency (cpd)^3^	Velocity (°/s)
5	(1.8, 1)	CW^1^	0.04	0
5	(1.8, 1)	CW	0.04	15
5	(1.8, 1)	CCW^2^	0.04	15
5	(1.8, 1)	CW	0.04	15
5	(1.8, 1)	CCW	0.04	15
5	(1.8, 1)	CW	0.04	15
5	(1.8, 1)	CCW	0.04	15
5	(1.8, 1)	CW	0.04	15
5	(1.8, 1)	CCW	0.04	15
5	(1.8, 1)	CW	0.04	15
5	(1.8, 1)	CCW	0.04	15
5	(1.8, 1)	CW	0.04	15
5	(1.8, 1)	CCW	0.04	15
60	(1.8, 1)	CW	0.04	15
60	(1.8, 1)	CCW	0.04	15

^1^Clockwise; ^2^Counter-clockwise; ^3^cycles per degree.

### Experimental procedure

**Figure 5:**
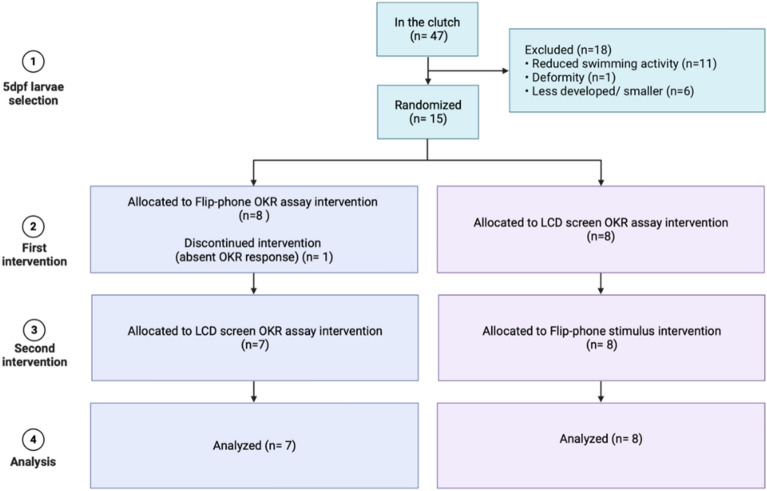
Flowchart outlining the experimental procedure, from selection to analysis. (1) Zebrafish larvae at 5 days post fertilisation (5 dpf) were randomly selected (n = 15) from a clutch. (2) Each larva is randomly assigned to either the flip-phone-based OKR assay or the traditional LCD-based OKR assay. (3) Following the completion of the first trial, each larva 'crossed over' to undergo the alternative assay. This cross-over approach is similar to a randomized cross-over clinical trial method. (4) Statistical analysis is conducted to compare results from both assays for each larva.

A total of 15 5 dpf zebrafish larvae were tested between 1 and 4 p.m. Each larva was exposed to both the LCD and flip-phone assays in a randomized sequence to minimize any potential systematic biases associated with time or fatigue. Eye movements were recorded and compared (Fig. 5).

### Eye movement recording and data analysis

Eye movement recordings were obtained using a Leica S9i digital microscope using the Leica Application Suite X (LAS X) software (version 5.0). Previously described MATLAB based software, *OKRtrack*^14^ was used to derive eye rotations which was subsequently analysed using custom scripts in Python (version 3.9). The eye movement plots were visualised using Matplotlib package (3.7.1) and slow phase eye velocity was derived from the traces. Briefly this consisted of finding peaks and troughs using second derivative-based algorithm and deriving velocity using the least-squares fit between peak and trough of the eye rotation data (Fig. 2). To enhance the accuracy of our measurements, the eye rotation data were filtered using a boxcar (moving average) method. This step helped to smooth out noise and fluctuations in the data, providing a clearer signal for analysis. The script identified different phases of eye movement, including slow and fast phases, by analysing changes in eye velocity. Specific thresholds were set to differentiate between these phases. Finally, the average slow-phase velocity was derived from the trial and used for subsequent statistical analyses.

### Statistical analysis

In this study, our main aim was to compare paired slow phase eye velocity obtained from two methods of projecting optokinetic stimuli, mobile phone and LCD screen, in order to investigate their similarity. We used IBM SPPS statistics version 28.0.0^20^ to analyse the data for normal distribution and calculate correlation coefficients for the paired measurements, and used Bland–Altman plots to assess agreement between two methods, providing insights into potential systematic biases (such as equipment calibration errors or non-uniform stimulus presentation) and the overall variability of differences^21,22^.

We conducted a sample size calculation for our method comparison study employing the Bland–Altman plot. Based on our preliminary study, we derived the following parameters: an anticipated mean difference of 0.60°/s, an expected standard deviation (SD) of differences of 0.10°/s, and a maximum allowable difference of 1°/s between the methods under consideration. By setting the Type I error (α) at 0.05 and the Type II error (β) at 0.10, we determined that a minimum of 12 pairs of measurements would be necessary to achieve statistically meaningful results.

### Supplementary Information

Supplementary Information.

## Data Availability

All slow phase velocity data derived from the experiments are all presented within the manuscript (Fig. 4).
